# 1837. Evaluation of Disparities in Beta-Lactam Allergies and Subsequent Antibiotic Use in Outpatients with Acute Bacterial Skin and Skin Structure Infections

**DOI:** 10.1093/ofid/ofad500.1666

**Published:** 2023-11-27

**Authors:** Ronald G Hall, Chip Shaw, Travis Cole, Carlos Alvarez

**Affiliations:** Jerry H. Hodge School of Pharmacy, Texas Tech University, Dallas Campus, Dallas, Texas, Dallas, Texas; Texas Tech UHSC, Lubbock, Texas; Texas Tech UHSC, Lubbock, Texas; Texas Tech UHSC, Lubbock, Texas

## Abstract

**Background:**

Black patients with skin and soft tissue infections are more likely to have a documented penicillin allergy and use of non-beta-lactams than white patients. We sought to compare the prevalence and risk factors for documented beta-lactam allergy (D-BLA) among racial and ethnic minorities (REM) and white patients and beta-lactam (BL) use for outpatient Acute Bacterial Skin and Skin Structure Infections (ABSSSIs).

**Methods:**

We conducted a cohort study of adult outpatients (18-90 years) seen by the Texas Tech University Health Science Center Physicians group (2012-2021). All patients had an ICD-9 or ICD-10 code for an ABSSSI in the primary or secondary position. Descriptive statistics were reported. Logistic regression models were used to determine which factors were statistically associated with D-BLA and which factors were associated with BL use for the ABSSSI. Multivariable (MV) analysis results were reported as odds ratios (OR) and 95% confidence intervals (95%CI). All analyses were conducted in R.

**Results:**

REM represented 38% of the 21,673 patients in the cohort. A D-BLA was present for 18% percent of all patients and was higher in white patients than REM (22 vs 13%). Factors associated with D-BLA on MV analysis of the total cohort were age (OR 1.01 per year, 95%CI 1.01-1.01), Medicaid (OR 1.18, 95%CI 1.04-1.34), or Medicare (OR 1.17, 95%CI 1.05-1.31). Factors associated with a lower likelihood of D-BLA on MV analysis of the entire cohort were REM (OR 0.55, 95%CI 0.51-0.60) and male sex (OR 0.56, 95%CI 0.52-0.60). Regarding BL use, white patients were less likely to receive a BL than REM (26 vs 30%). Factors associated with a reduced likelihood of BL use on MV analysis of the total cohort were D-BLA (OR 0.60, 95%CI, 0.54-0.67), Medicaid (OR 0.83, 95%CI 0.74-0.93), or Medicare (OR 0.64, 95%CI 0.58-0.70). Factors associated with BL use on MV analysis of the entire cohort were REM (OR 1.20, 95%CI 1.12-1.28), male sex (OR 1.45, 95%CI 1.36-1.54), or self-pay (OR 1.52, 95%CI 1.37-1.68). The interaction term between REM and D-BLA was not significant for BL use.

**Conclusion:**

REM were at a reduced risk of a D-BLA in our cohort and were more likely to receive a BL for ABSSSIs. Payor status and sex also significantly impacted rates of D-BLA and BL use.

**Disclosures:**

**Ronald G. Hall, II, PharmD, MSCS**, Merck: Grant/Research Support **Carlos Alvarez, PharmD, MSc, MSCS**, Boehringer Ingelheim: Grant/Research Support|Bristol Myer Squibb: Grant/Research Support|Merck: Grant/Research Support

